# Quaternion-Based Gesture Recognition Using Wireless Wearable Motion Capture Sensors

**DOI:** 10.3390/s16050605

**Published:** 2016-04-28

**Authors:** Shamir Alavi, Dennis Arsenault, Anthony Whitehead

**Affiliations:** 1 Systems and Computer Engineering, Carleton University, Ottawa, ON K1S 5B6, Canada; shamiralavi@cmail.carleton.ca; 2 School of Information Technology, Carleton University, Ottawa, ON K1S 5B6, Canada; dennisarsenault@cmail.carleton.ca

**Keywords:** gesture recognition, wearable sensors, quaternions, pattern analysis, machine learning, support vector machines, artificial neural networks

## Abstract

This work presents the development and implementation of a unified multi-sensor human motion capture and gesture recognition system that can distinguish between and classify six different gestures. Data was collected from eleven participants using a subset of five wireless motion sensors (inertial measurement units) attached to their arms and upper body from a complete motion capture system. We compare Support Vector Machines and Artificial Neural Networks on the same dataset under two different scenarios and evaluate the results. Our study indicates that near perfect classification accuracies are achievable for small gestures and that the speed of classification is sufficient to allow interactivity. However, such accuracies are more difficult to obtain when a participant does not participate in training, indicating that more work needs to be done in this area to create a system that can be used by the general population.

## 1. Introduction

Increasing our work efficiency while being able to perform tasks accurately is a problem people have been trying to solve for years. The physical nature of our body limits us from being consistently efficient in performing long, repetitive tasks. To overcome this problem, scientists and engineers started developing assistive technologies which would recognize and imitate human gestures to perform tasks that we consider difficult or strenuous. As a result, developing systems that can capture human motion and recognize different gestures simultaneously is important.

Gestures are physical movements of different parts of the body that are expressive and meaningful to human beings. We perform gestures to convey information or to interact with the environment. Gesture recognition has a wide variety of applications that include, but is not restricted to, developing aids for the hearing impaired, recognizing sign language, navigating in virtual environments, and automation of manufacturing tasks, *etc*. [1]. There are various techniques that can be used to recognize gestures, ranging from using mathematical models based on the Hidden Markov Model [2] and Markov Chains [1] to applying computer vision-based approaches [1,3], by using data gloves [4] and accelerometers [5,6], or using a combination of any of the above [1]. Gestures can be hand and arm gestures, head and face gestures or full body movements. It is important to note that a gesture is a small movement and tasks or activities can be considered as a series of gestures performed in sequence.

The work presented in this paper is a continuation of work that used wearable IMU (Inertial Measurement Unit) sensors to implement a full body motion capture system in virtual reality [7]. This work suggests that motion capture and gesture recognition can be combined into a single system that allows for fully body gesture capture and recognition and will facilitate grammar-based activity recognition, all possible at interactive rates. Finally, this work also demonstrates two key novel elements: one, that the gesture recognition system does not require a kinematic model to achieve good results and; two, the human being does not have to be overly instrumented to achieve reasonable results.

## 2. Related Works

A lot of work on gesture recognition can be found in the current literature which incorporates the use of different types of sensors and models for several real life and virtual applications. They cover both IMU sensor-based applications as well as vision-based recognition systems. Gesture recognition using virtual reality interfaces became more prominent with the invention of systems like Microsoft’s Kinect [8], the Nintendo Wii, Oculus Rift, *etc*. In [9], a hidden Markov model-based training and recognition algorithm was used on data collected from a Wii controller to develop a gesture recognition system.

Vision-based gesture recognition has been on the rise since we started developing better cameras, image and video compression technologies, in consort with faster processors and GPUs (Graphics Processing Units). These systems cover several application areas such as surveillance, detection, control and other analyses of captured motion data. Using a multiple sensor based approach, Lementec *et al*. [10] presented a gesture recognition algorithm using Euler angles. Their work is part of a control system for an Unmanned Aerial Vehicle (UAV). A wide array of vision-based approaches exist and the reader may explore [3,11] for details. The problems with vision-based systems are their high computational expense as they incorporate the use of GPUs, the limited range of use created by the camera viewing volume, and a large number of cameras required to cover large spaces.

Many gesture recognition systems exist which use a specific part or parts of the body to recognize gestures using a limited number of sensors. This has become more apparent in recent work as a result of the increasing popularity of wearable fitness devices. uWave is a gesture recognition system that uses gesture-based interactions from a single three-axis accelerometer [12]. It requires a single training sample for each pattern and allows users to define their own personal gestures. An automatic hand gesture recognition system has also been developed for use in Augmented Reality [13]. It is able to differentiate between static and dynamic gestures.

Zhu *et al*. [14] created a smart assisted living (SAIL) system that can aid the elderly and the disabled using a human-robot interaction (HRI) technology. They studied hand gesture recognition and daily activity recognition using artificial neural networks. For daily activity recognition, they developed a multi-sensor fusion system.

The examination in [15] uses only an eight-sensor system *versus* our ten, and in that configuration, it cannot do effective motion capture. The inverse kinematic elements will not work if major bones are missed during motion capture. Moreover, their recognition rates fall around the 80% mark. The work in [16] is an old project that was intended to assist developers in developing applications without having to know much about machine learning systems. The recognition rates for this project allow any system which is built with it, to recognize only the simplest of gestures (for example, left *versus* right-hand gestures using an accelerometer). In the study performed in [17], the sensor is on the device that the person is interacting with, and not being worn on the body. Furthermore, the gestures or activities selected could be classified with an accelerometer alone given the simple nature of the gestures. The work in [18], while interesting, also takes on the simplest of activities (Tai Chi). Because of the slow motion, much can be done by orientation sensors rather than pure body pose. The authors do not mention anything about runtime. With the work being about Dynamic Time Warping as well, it seems like they were also working on pre-segmented data. Overall, much work has been done evaluating different methods of gesture recognition with IMU sensors for various subsets of the body including the arm [10,19,20], the leg [21], the torso [22] and the head [23,24]. In these cases, a smaller number of IMU sensors were used due to the localized focus on a specific body part. In all of these cases, the subset of sensors to recognize gestures on a single limb eliminates the opportunity for full body motion capture.

Some full-body capture, recognition or interaction systems can be found in the current literature, such as ALIVE [25]. A couple of view-invariant full-body gesture recognition systems are described in [26,27], but these are vision-based systems. On the other hand, references [28,29,30] describe Microsoft Kinect-based full-body recognition. These systems offer little mobility as the Kinect sensor needs to be kept in place to capture data, and the Kinect cannot be used outdoors. A gesture recognition system to interact with a robot has been introduced in [17], but the sensors are in the robot, not on the body of a human being.

The system that most closely resembles our work is the OPPORTUNITY project [31], a full-body complex activity recognition system. However, the authors used 72 sensors of 10 different modalities (including IMU sensors) to capture data from twelve subjects. It should be noted that with so many sensors in the system, it is impossible to determine the generalization capabilities until a test dataset grows immensely large. The OPPORTUNITY project not only instruments the human very highly, it also instruments the environment and objects that are interacted with. Thus, any activity that uses the instrumented fork, for example, is clearly separable from the rest of the activities to be detected. This allows for a simple hierarchical classification system, such as a decision tree, as a first step. The authors use standard pattern classifiers such as k-NN, NCC, LDA and QDA in [32,33] to evaluate and benchmark the activity dataset. They use only mean and/or variance as features, which is understandable because the data from 72 sensors are already quite descriptive for classification purposes. The overlap in IMU sensors between this work and our own consists only of five IMU sensors, four located on the upper and lower arms. The fifth IMU sensor used is on the back, whereas ours is placed on the abdomen, which aside from the obvious orientation issues, should be similar. Our sensors are a subset of the IMU sensors used in the OPPORTUNITY project, and ours use only gyro and accelerometer data processed through a built-in sensor fusion algorithm that produces a quaternion value. Moreover, we don’t assume a kinematic model or sensor hierarchy in the gesture recognition portion of this work.

There are not many wearable gesture recognition datasets available publicly, making direct comparisons of methods difficult. As seen from [34], most of the full-body datasets are Kinect-based apart from a few, such as the OPPORTUNITY dataset mentioned above. Although we are doing an upper-body gesture recognition, the outcome from our study will be the foundation for modeling a wearable sensor-based full-body gesture recognition system. However, the recent survey by LaViola Jr. [35] examines the results of many different 3D gesture recognitions systems and ours is comparably accurate.

Our contributions to the current state-of-the-art include:

Extracting five feature descriptors including velocity and angular velocity from quaternions which are very good at representing rotations as compared to Euler angle or matrix representations [36] and eliminating gimbal lock.

Using a limited number of sensors to preserve as much generalization in the data as possible but cover as many of the major movements of the upper body.

Presenting an interactive recognition rate that will allow for more complex activity recognition at interactive rates.

Presenting results that generalize to the population, *i.e.*, where the test users dataset is not included in training.

This work also suggests that to achieve reasonable recognition for smaller gestures that can allow non-traditional interaction with systems, the human does not need to be so highly instrumented, nor does the environment need to be instrumented.

This work exhibits the effect of velocity on recognition rates and suggests that velocity and acceleration features should only be included when speed of the gesture is an important performance consideration. E.g. training applications.

We make our dataset publicly available (see Supplementary Materials).

## 3. Background

In this study, we used Body Area Sensors to capture motion data for different gestures in the form of quaternions. We used the collected data as input to a gesture recognition system built around two classical pattern classification algorithms, namely, Support Vector Machines (SVM) and Artificial Neural Networks (ANN). We followed standard experimental methodologies that are used to evaluate such classification systems.

### 3.1. Body Area Sensors

**Figure 1:**
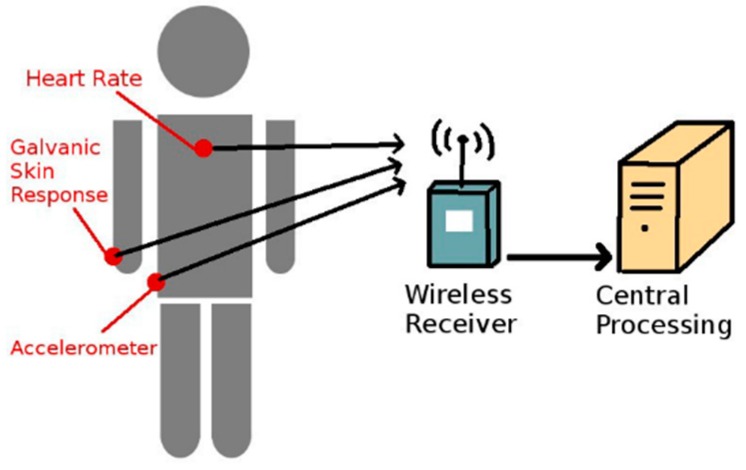
A theoretical Body Area Sensor Network (BASN)**.**

Body area sensors determine the current physical state or changes in such states of an individual. Typically, these are used in a sensor network called Body Area Sensor Network (BASN) [37]. These sensors are typically worn on the body. In a few special cases, they may also be implanted within the body. Figure 1 shows a basic design of such a network and how information is transferred from an individual to the data processing unit.

With recent technological advances, sensors have become much smaller. The development of Micro-Electro-Mechanical Systems (MEMS) [37] have enabled sensors to generate data faster, operate on batteries, communicate wirelessly and provide easier wearability. Sensors have applications in a wide variety of fields that can be divided into two principle categories—physiological and mechanical. Our interest in this work lies in three different mechanical sensors which are accelerometers, gyroscopes, and magnetometers.

An accelerometer can measure acceleration that occurs along a device’s axis. 3-axis accelerometers are used so that acceleration can be measured in any direction. They can provide information on their angle of inclination with respect to downward direction by sensing the acceleration due to gravity. However, these sensors are unable to distinguish between gravitational force and actual acceleration [38]. They suffer most from noise in their readings.

Gyroscopes are used to detect angular velocities. However, they are unable to determine their absolute orientation and suffer from drift issues [39]. Drift adds small angular velocities even when the device is completely stationary. Over time, these accumulate and affect the overall sensor reading.

Magnetometers are used to discern the direction of Earth’s local magnetic field. They use Earth’s field as a reference direction. As Earth’s magnetic field is quite weak, one thing to be careful about while using magnetometers is to keep them away from nearby metallic objects to prevent magnetic interference.

Using accelerometers, gyroscopes and magnetometers together can produce better measurements than that possible with any of these sensors individually. This combination produces more accurate information about the motion by using a sensor fusion algorithm [40]. The device is often collectively called an Inertial Measurement Unit (IMU) [41].

### 3.2. Coordinate System: Quaternions

The sensors that we use in this work output their orientations in the form of quaternions. A quaternion is comprised of a scalar component and a vector component in complex space. This representation can be seen in the following equations:

(1) $$q=\left(r,\vec{v}\right)=(q_{r},\;q_{x}\hat{i},q_{y}\hat{j},q_{z}\hat{k})$$

Here $\hat{i},\;\hat{j}$ and $\hat{k}$ are complex orthogonal basis vectors which makes quaternion multiplication a non-commutative operation. More details can be found in [42]. All quaternions that represent a 3D rotation are represented by unit quaternions. A unit quaternion has a magnitude of 1 which means the absolute value or norm [43] of “*q*” would be:

(2) $$\left|q\right|=\;\sqrt{q_{r}{}^{2}+\;q_{x}{}^{2}+\;q_{y}{}^{2}+\;q_{z}{}^{2}}=1$$

Quaternions are extremely efficient at representing rotational and orientation information. A rotation of angle θ about a unit axes $\vec{n}$ can be represented as:

(3) $$q_{1}=\left(cos\frac{\theta }{2},\;\vec{n}\;sin\frac{\theta }{2}\right)$$

To rotate the current orientation $q_{0}$ by the amount specified by $q_{1}$, we multiply it by $q_{1}$:

(4) $$q_{2}=\;q_{1}q_{0}$$

This quaternion $q_{2}$ is equivalent to rotating a rigid body (in a current orientation described by $q_{0}$) by a rotation described by $\;q_{1}$. A series of rotations can therefore be represented by a series of quaternion multiplications. It is a very efficient method of computing and representing a series of rotations. Quaternions can be a powerful form of representing rotations over other forms of representation. One of the biggest benefits of using quaternions is that they do not suffer from issues such as gimbal lock that methods like Euler angles do. Moreover, they are very computationally efficient because they do not require the calculations of many trigonometric functions. On the contrary, they suffer from being more conceptually difficult to understand and more abstract to visualize.

### 3.3. Pattern Classification and Feature Extraction

Pattern classification involves the use of (pattern) classifiers to distinguish among different, interesting patterns inherent in a dataset. In the broadest sense, it employs learning methods to find meaningful patterns in the data [44]. Learning refers to the use of some form of algorithm to reduce error on the training portion of the dataset. It comes in several forms such as supervised learning, semi-supervised learning, unsupervised learning, representation learning *etc*. In this study, we used two supervised learning algorithms—Support Vector Machines (SVM) and Artificial Neural Networks (ANN), as classifiers for our gesture recognition system. For a much more detailed discussion on SVM and ANN, the reader can refer to [45,46,47,48].

Data that exhibits good class separation is a general requirement for classifiers to perform well during the classification process [49]. This can be achieved by extracting and/or selecting meaningful features from the raw dataset. On the other hand, reducing the dimensionality of a feature set enables faster runtime, hopefully without compromising classification accuracy [50].

Principal Component Analysis or PCA, among many others, is a dimensionality reduction technique that is most commonly used in pattern classification. PCA projects multi-dimensional data into a lower dimensional space or hyperspace but preserves necessary variations in the dataset. A broader explanation about PCA can be found in [51].

## 4. An IMU Motion Tracking System

**Figure 2:**
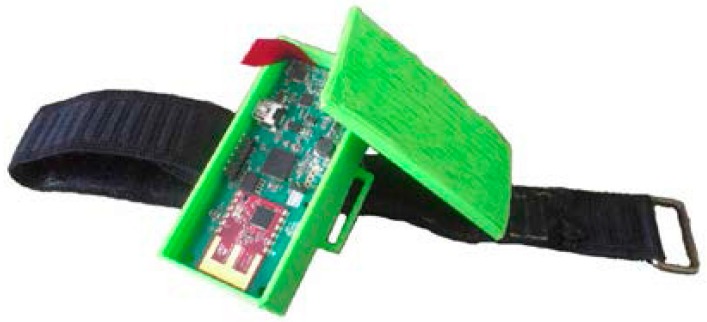
IMU sensor inside its 3D printed case.

The system proposed in [52] uses the IMU sensors mentioned in Section 3.1 to track the pose and motion of an individual. 3D printed cases were made for the sensors so that each one can be fit inside a case and attached to the body using Velcro straps as shown in Figure 2. The system is comprised of 10 sensors attached to the body.

**Figure 3:**
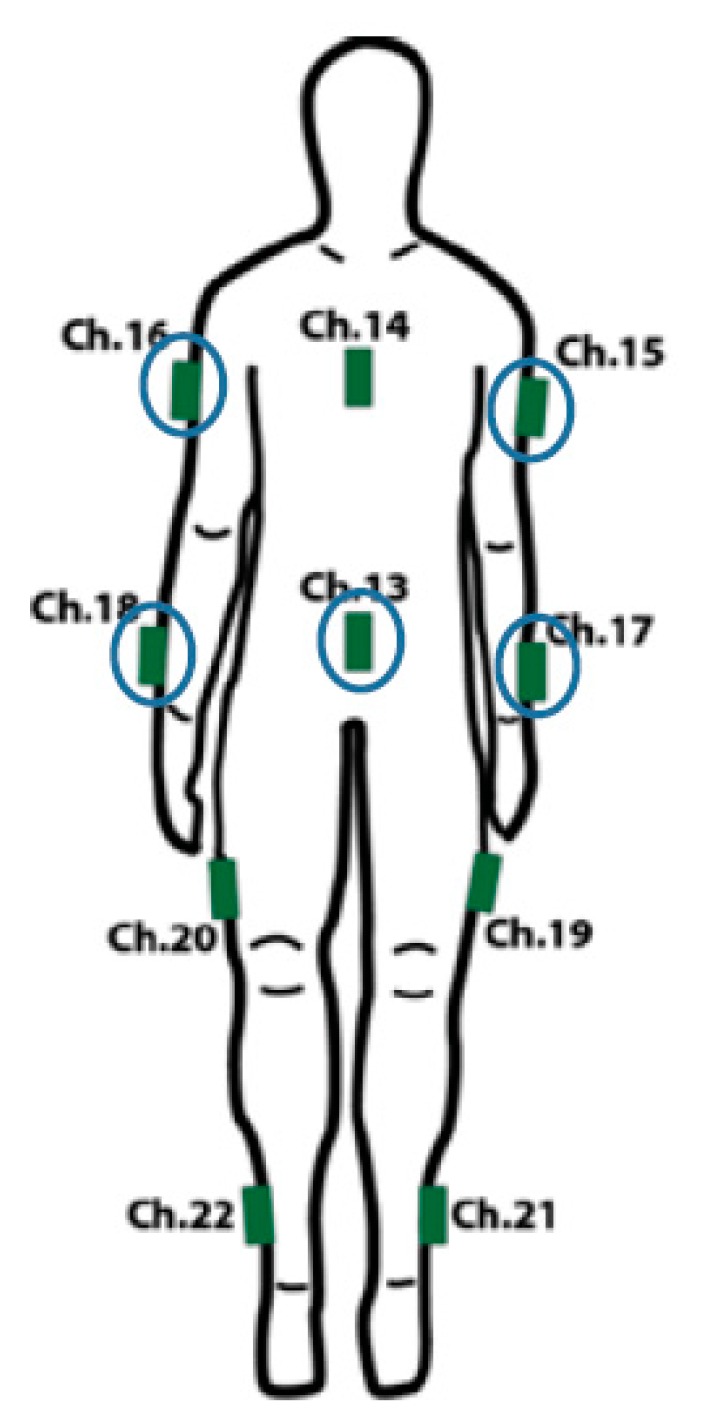
Sensor placements on the body for the motion capture system (green) and the subset of sensors used in the gesture recognition portion of this work (circled)

One sensor is assigned to each body segment, hereafter referred to as bone. The sensors are programmed to operate on their own individual frequencies. The placement of the sensors along with their corresponding frequency channels is shown in Figure 3.

A character model was imported into the Unity game engine [53] to animate based on the sensor data. The bones were modeled as separate objects to allow greater positional control.

### 4.1. Sensor and Bone Orientation

**Figure 4:**
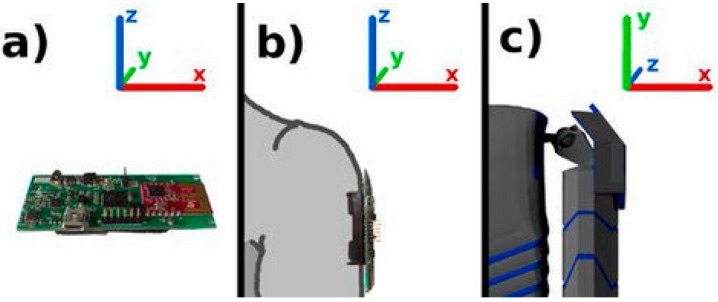
(**a**) Global orientation of sensor on startup; (**b**) Global reference frame of the sensor on the right upper arm; (**c**) Character’s arm orientation in Unity's axis frame.

Upon startup, the sensors generate their own global reference frame in quaternion orientations to represent rotations with respect to the initial reference frame. Figure 4 shows the initial reference generated by a sensor, the corresponding initial orientations of the right upper arm of the user, and the character’s arm orientation in Unity’s axis frame.

To resolve these issues while mapping the player's motions to the character, the quaternion was converted from the sensor's global frame to the Unity frame in order for the rotation directions to match correctly. The offset of the quaternions was also changed so that the orientation of the character's bones matched those of the user at the start of the program.

### 4.2. Coordinate System Transfer

A change in system handedness is required due to the sensors right-handedness and Unity`s left-handed frame. To fix this problem, any rotation value of θ was changed to −θ by taking advantage of the even/odd nature of the sine and cosine functions. The final mapping from raw sensor quaternion $q_{0}$ to the Unity re-mapped quaternion $q^{\prime }{}_{0}$ is given by:

$$q_{0}\;=\left(q_{w}\;q_{x}\;q_{y}\;q_{z}\right)$$

(5) $$q^{\prime }{}_{0}=\left(q_{w},\;-q_{x},\;-q_{z},-q_{y}\right)$$

The i, j, k bases are omitted for easier readability since it is implicit based on their position in the vector part of any unit quaternion.

### 4.3. Initial Quaternion Offset

The orientation of the onscreen character's bone must match properly to that of the player’s. This was accomplished by making the player stand in the matching attention pose of the character as shown in Figure 3. Now, to represent the rotations in quaternion form, $q^{\prime }{}_{0}\;$ was defined as the sensor's quaternion output while the user in in the pose shown in Figure 3, and $q_{t}{}^{\prime }\;$ as any other valid orientation at a later time t. As a result, there exists another rotation $q^{\prime }{}_{1}$ that takes $q^{\prime }{}_{0}$ (initial pose) to $q^{\prime }{}_{t}$ (current pose):

(6) $$q^{\prime }{}_{t}=\;q^{\prime }{}_{1}q^{\prime }{}_{0}$$

This rotation, $q^{\prime }{}_{1}$ is the rotation that the bones use for their orientation. To isolate, we simply multiply each side of the equation by the complex conjugate of $\;q^{\prime }{}_{0}$. Thus at any later time t, the bones would rotate by:

(7) $$q^{\prime }{}_{1}=\;q^{\prime }{}_{t}q^{\prime *}{}_{0}$$

Here, * refers to the complex conjugate of the quaternion $\;q^{\prime }{}_{0}$. This resets the starting orientation of each sensor to be its orientation relative to when the motion capture system starts (at the pose in Figure 3), rather than when the sensors are turned on.

The bones of the virtual character obtain a rotational offset ($q_{e})$ once the character is imported from the 3D modeling program to the game engine. Therefore, one additional offset is required for each bone to neutralize the default offset amount. This rotation must be included in the calculation so that the virtual character’s bones starts in the proper attention pose (Figure 3) before rotating to match that of the player’s current pose. Thus, the final quaternion representation of the total rotation is given by:

(8) $$q^{\prime }{}_{1}=\;q^{\prime }{}_{t}q^{\prime *}{}_{0}q_{e}$$

### 4.4. Sensor Calibration

**Figure 5:**
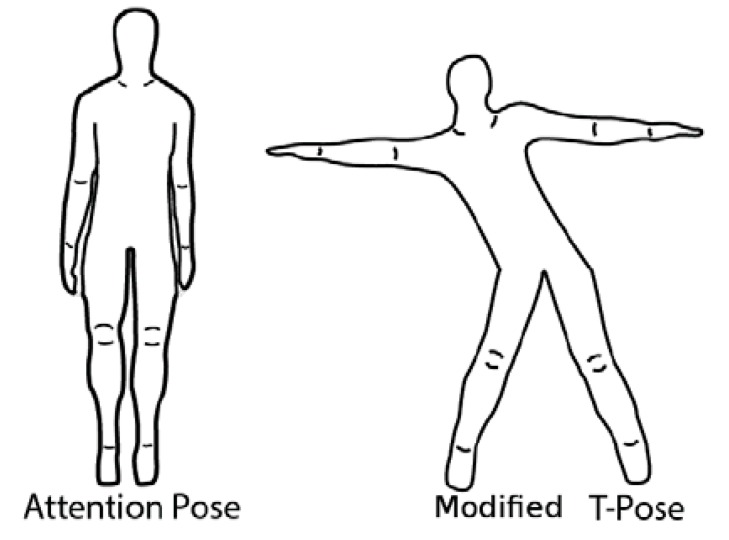
Poses used to perform the calibration routine.

The sensors needed to be calibrated to avoid their strong reliance on the startup position and to reduce sensor drift [54] about their vertical axes. These issues cause axes misalignment which results in the character's on-screen motion not exactly matching that of the player’s. The calibration routine has two steps: the first is the initial attention pose and the second is the modified T-pose (Figure 5). A few seconds of transition time between each pose is allowed to complete the calibration.

The following equation determines which axis the sensor rotated around between the attention and modified T-pose:

$$v_{1}=\left(q^{\prime }{}_{T}q^{\prime *}{}_{A}\right)v_{0}{\left(q^{\prime }{}_{T}q^{\prime *}{}_{A}\right)}^{*}$$

(9) $$=\;q^{\prime }{}_{T}q^{\prime *}{}_{A}v_{0}q^{\prime }{}_{A}q^{\prime *}{}_{T}$$

**Figure 6:**
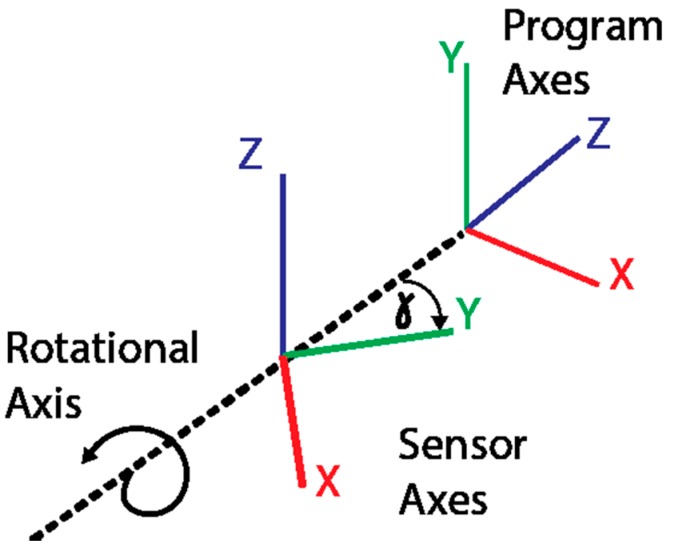
Different orientations of the rotation axis between the attention pose and the T-pose.

Here, $q^{\prime }{}_{A}\;$ is the sensor’s reading in the attention pose, $q^{\prime }{}_{T}\;$ is the reading in the modified T-pose, $v_{1}$ is the new vector resulting from rotating the initial orientation $v_{0}$ through the same rotation the sensor underwent from the attention pose to the modified T-pose. Figure 6 shows the relative difference in orientation of the reference frames of the sensor axes and Unity’s program axes for the two poses.

The rotation angle of drift γ, which is the angle between the ideal and current sensor’s reference frames, is determined by:

(10) $$\gamma =\mathrm{atan}(\frac{v_{2x}}{v_{2z}})$$

where $v_{3}$ is the cross product between $v_{1}\;$ and $v_{0}\;$ vectors as seen in Equation (11):

(11) $$v_{3}=\;v_{1}\;\times \;v_{0}$$

Using the drift (γ) calculated for each sensor, the different reference frames are aligned using the previously determined mapping between the raw sensor quaternions and the Unity quaternions from Equation (5). This mapping was rotated about the vertical axis after sensor calibration to align the sensor's reported y-axis properly with Unity's z-axis. The newly calibrated mapping, $q_{cal}\;$ is given by:

(12) $$q_{cal}=(q_{w},\;-\left(q_{w}\cos\left(-\gamma \right)+q_{y}\sin\left(-\gamma \right)\right),\;-q_{z},\;-\left(-q_{x}\sin\left(-\gamma \right)+q_{y}\cos\left(-\gamma \right)\right))$$

Here,  q  is the sensor’s quaternion output. $q_{cal}$ is the calibrated quaternion that we use for the remaining calculations.

### 4.5. Skeletal Model

By default, Unity takes care of positioning all of the bones. However, this resulted in the loss of control of several factors, including the order that the bones were updated. This, in turn, led to the poorer performance and a lower quality tracking result. To overcome this problem, a specific skeletal model was created to handle the positioning of all the bones. The positioning of the bone base and tip positions were redefined and new calculations for the skeletal walking design were developed. More details on this can be found in [52].

## 5. Gesture Recognition Using Multiple Sensors

With the technical setup in place for a wearable motion capture system, we designed a comprehensive gesture recognition system. The model was built to classify among six different gestures performed by humans. We followed standard experimental methodologies to run the experiment and analyze the results using SVMs and ANNs.

### 5.1. Features

We calculated variance, range, velocity, angular velocity and covariance from the dataset. As the data was collected from a set of five sensors, every feature label contains the serial number of the sensor that is relevant to the feature. A feature labeled as “var_15_qx” refers to variances calculated using the $q_{x}\;$ vector from Sensor 15. We call these types of features “Single Sensor-based Features” because each of these features are calculated by using data from one sensor at a time. On the other hand, a feature labelled as “cov_15_16_qx” refers to the covariance of Sensor 15 and Sensor 16’s output for the quaternion element$\;q_{x}$. Variance, Range, Velocity and Angular Velocity are the four single sensor-based features used in this study whereas Covariance is the only multiple sensor-based feature that has been used here. The entire feature set has 115 features. Variance, range and, covariance are simple statistical measurements of the data whereas velocity and angular velocity are physical properties extracted from the dataset.

Velocity, by definition, is the distance traveled over time towards a specific direction, *i.e.*, the speed of an object towards the direction it is traveling. We considered this as an important feature because every gesture is unique and, therefore, should show varying degrees of velocity. Moreover, it is expected that different participants will perform gestures at different speeds. We calculated distance summing over the Euclidean distances of each consecutive data points in every sample. Thus, velocity was calculated in the following manner:

$$velocity=\;\frac{total\;distance\;covered}{total\;time\;spent\;to\;cover\;the\;distance}$$

(13) $$=\;\frac{\sum_{i=1}^{n}Euclidean\;distance\;(x_{i+1},\;x_{i})}{time}$$

To calculate time, we used the following equation:

(14) $$\left(elapsed\right)\;time=\;\frac{number\;of\;datapoints}{sensor\;frequency}$$

In Equations (13) and (14), $x_{i}$ is the value of data point at the i-th index of the sample, n is the number of data points in the sample and sensor frequency is 110 Hz.

Angular velocity is the rate of change of angular displacement of an object about its axis of rotation [55]. In other words, it is the rate of change of angular positions of a rotating body. This feature gives us positional information of the active limbs in 3-D space. The rationale behind using this as one of the features is similar to that of using velocity. It is calculated in two steps—the first step is to convert the quaternions to Euler angles and the next step is to calculate the angular velocities from these angles. From the different parameterizations that are possible to convert quaternions to Euler angles [56], we chose to use:

(15) $$\alpha =atan(\frac{2(q_{w}q_{x}+\;q_{y}q_{z})}{1-2(q_{x}{}^{2}+\;q_{y}{}^{2})})$$

(16) $$\beta =asin(2(q_{w}q_{y}-\;q_{x}q_{z}))$$

(17) $$\gamma =atan(\frac{2(q_{w}q_{z}+\;q_{x}q_{y})}{1-2(q_{y}{}^{2}+\;q_{z}{}^{2})})$$

where −π< α≤ π; $-\frac{\pi }{2}<\;\beta \leq \;\frac{\pi }{2}$; −π< γ ≤ π; $q_{w},q_{x},\;q_{y},\;q_{z}\;$ constitute a unit quaternion q.

We can calculate the angular velocity vectors Precession, Nutation, and Spin from Equations (15)–(17) as follows:

(18) $$precession=\;\dot{\alpha }\sin\left(\gamma \right)\sin\left(\beta \right)+\dot{\beta }\cos\left(\gamma \right)$$

(19) $$nutation=\;\dot{\alpha }\cos\left(\gamma \right)\sin\left(\beta \right)-\;\dot{\beta }\sin\left(\gamma \right)$$

(20) $$spin=\;\dot{\alpha }\cos\left(\beta \right)+\;\dot{\gamma }$$

Here:

$$\dot{\alpha }=precession\;velocity$$

(21) $$\frac{\sum_{i=1}^{n}Euclidean\;distance\;(\alpha_{i+1},\;\alpha_{i})}{time}$$

Similarly:

(22) $$\dot{\beta }=\;\frac{\sum_{i=1}^{n}Euclidean\;distance\;(\beta_{i+1},\;\beta_{i})}{time}$$

(23) $$\dot{\gamma }=\;\frac{\sum_{i=1}^{n}Euclidean\;distance\;(\gamma_{i+1},\;\gamma_{i})}{time}$$

The parameter ‘time’ in Equations (21)–(23) is calculated in a similar manner as in Equation (14). Here, sensor frequency is 82.5 Hz, which is calculated after the conversion to Euler angles. We have 15 of these features.

### 5.2. Data Collection and Partitioning

**Figure 7:**
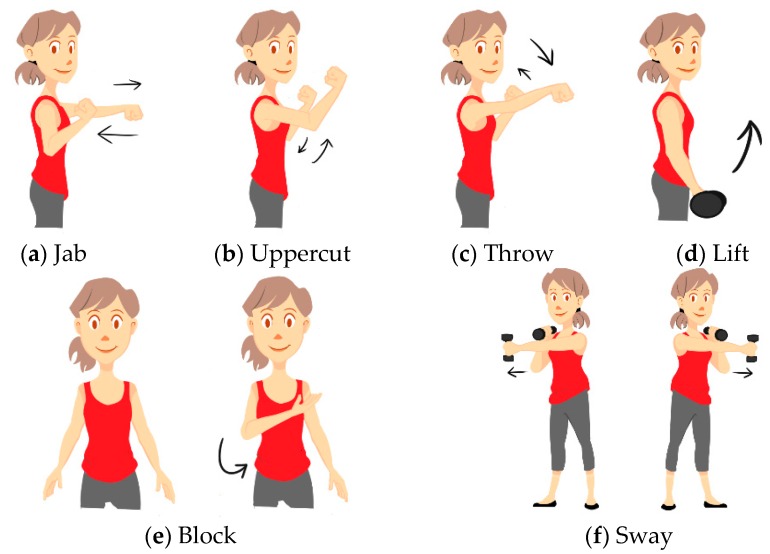
Gestures performed during each study. (**a**) Jab; (**b**) Uppercut; (**c**) Throw; (**d**) Lift; (**e**) Block; (**f**) Sway.

Data was gathered using a subset of sensors from the wearable motion capture system described previously. Two sensors were placed on both the arms and one on the upper abdomen (positions Ch. 13, 15, 16, 17 and 18 in Figure 3). We used six gestures in our study: Jab, Uppercut, Throw, Lift, Block and Sway (Figure 7). Jab, Uppercut, and Block are combat gestures. Throw, Lift and Sway can be related to other aspects of our daily lives in different ways.

We collected anonymous data from 11 participants, four females and seven males. Our raw dataset has around 20 samples per gesture from each participant. For each sample, participants were asked to perform the respective gesture for about five seconds using one of their hands while keeping the other hand still. They used their left and right hands alternatively for each sample which yielded in 50% data from the left hands (LH) and 50% from the right hands (RH) (not applicable for Sway). On average, they performed five instances of a gesture continuously within the timeframe. Thus, each of the five sensors in our setup collected about 100 instances (20 samples × 5 instances/sample) of every gesture per participant. This yielded in a total of about 600 instances per participant from each sensor.

While these numbers reflect the ideal scenario, problems like missing values and participants getting fatigued contributed to the discrepancy in the actual number of samples. From the raw dataset, we derived a Euler angle dataset. Both of these datasets were used to extract various features which are explained later in Section 5.4. The raw dataset consists of 1080 samples which contain a total of 2,959,765 coordinates. Table 1 shows the gesture-wise distribution of samples.

Using the raw and the Euler angle datasets, we have extracted 124,200 features points, in the form of 115 identical features for every sample in the dataset. To deal with missing values (due to sensor data loss, for example), we interpolate the value from the prior and post data values that were available.

**Figure 8:**
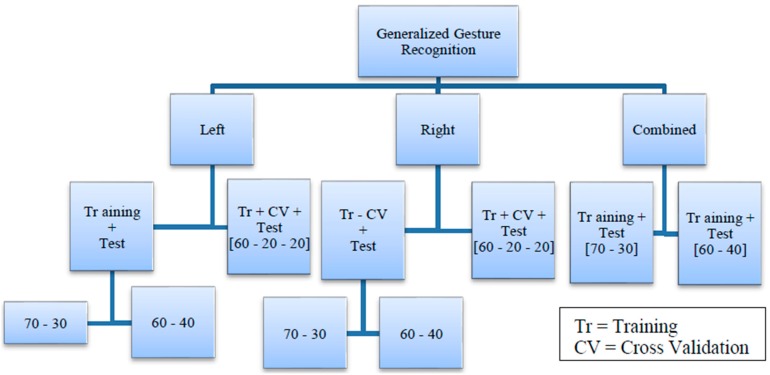
Dataset splits for Generalized Gesture Recognition.

We organized the data into two categories: Generalized Gesture Recognition and User Specific Gesture Recognition. The generalized dataset includes training and test set data from any participant without repetition. Here, we are not interested in the individual from whom the data is coming but only interested in the gestures. This rule applies to any of the training, cross-validation or test set under this category. The splits for this dataset is shown in Figure 8.

**Figure 9:**
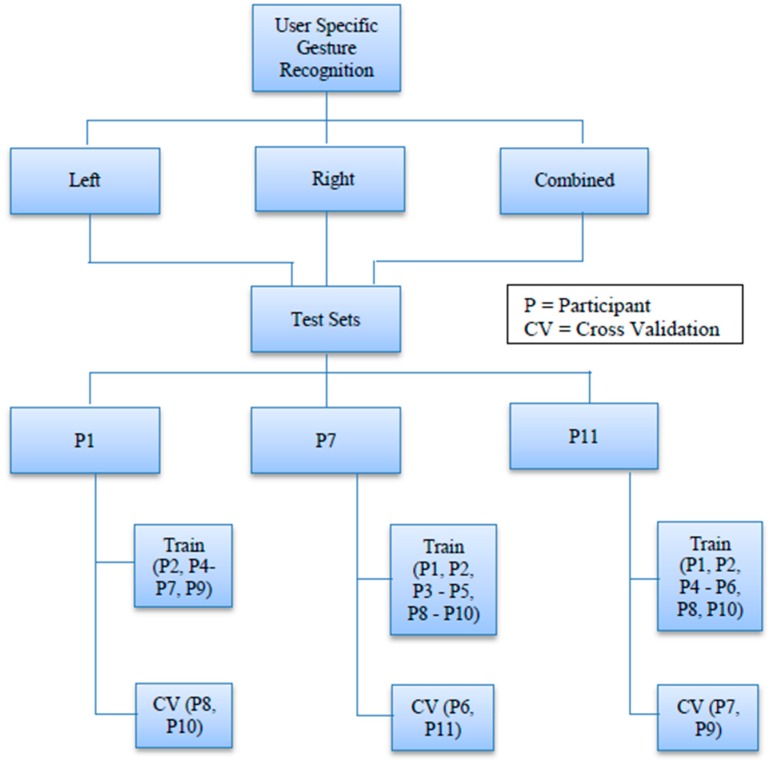
Dataset splits for User Specific Gesture Recognition.

Every user specific dataset comprises a test set that contains data from a specific individual within the participants. As a result, the corresponding training set does not contain any data from this individual. Figure 9 shows the data splits for this case.

We made three different test cases and none of them contains any common sample. For example, case P1 (Figure 9) has a test set that contains data from only participant 1 but case P7 has a test set that includes data from participant 7 only. This method tests whether our model is capable of recognizing a particular person’s gestures as opposed to recognizing any gesture in general, which is a probable reflection of a real life gesture recognition scenario where a user does not need to train in order to use the system. During the selection process of our test sets, we tried to maintain fairness by randomizing the sequence of the participant datasets before selecting the three mentioned above.

Both the categories have separate datasets corresponding to left-hand gestures, right-hand gestures and a combined dataset that includes data from both of these sets. Each dataset is divided into three parts: Training, Cross Validation and Test sets with proportions of 60% for training, 20% for cross-validation and 20% for testing. For the combined case, two different partitions were made with the proportions being 70%–30% and 60%–40% respectively for training and testing.

### 5.3. Data Preprocessing

After creating the partitions, we standardized the datasets so that they have zero mean and unit variance. Standardization (also called scaling) was done in batches such that the cross-validation and test sets have same standardized outputs as their corresponding training sets. It is very useful for classifiers like SVM and Neural Network. Scaling the data to fit into a smaller numeric range such as [0,1] or [−1, +1] lets all features contribute equally to the classification process. It can also make training faster because of the uniform, smaller range of the dataset [57]. This is very beneficial in training Artificial Neural Networks as it reduces its chances of getting stuck in local optima [58].

### 5.4. Classifier Setup and Initial Experiment

With the data prepared for the training phase, we performed a quick experiment to understand how the two classifiers, SVM and ANN would perform after being trained with scaled data. We also used the results from this step to tune the parameters of the classifiers. We ran this experiment for both the generalized and user specific case.

#### 5.4.1. SVM

Cost was set to 1.0 to 3.0 with increments of 0.5, kernel was set to linear. Varying the cost parameter over 1.0 did not yield any difference in the results. Therefore, we decided to use 1.0 later in our experiment as well. We used “linear kernel” because we have a large number of features. Non-linear kernels map data onto a higher dimensional space, which we do not require for our feature set.

#### 5.4.2. ANN

1 through 20 and “a” numbers of units in the hidden layer were tested where $a\;=\;\frac{\#\;of\;features\;+\;\#\;of\;classes\;}{2}=\;\frac{115+6}{2}\approx 60$, learning rate, α was set to 0.1, momentum, m was set to 0.2, epoch was set to 500, validation set size was set to 20% and validation threshold was set to 20.

**Figure 10:**
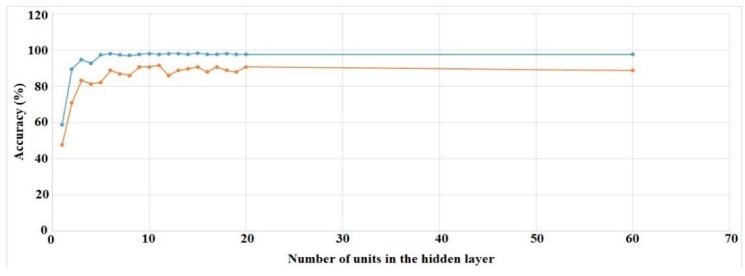
ANN training (blue) and validation set (red) accuracies for different number of units in the hidden layer (RH, generalized).

**Figure 11:**
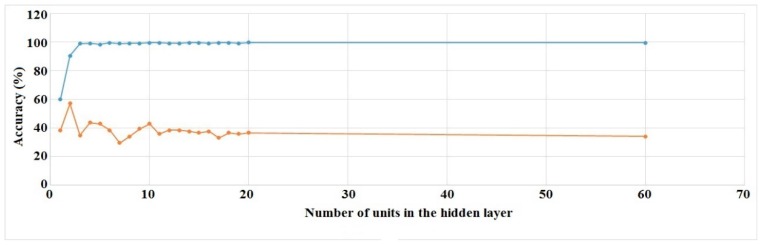
ANN training (blue) and validation set (red) accuracies for different number of units in the hidden layer (LH, user specific).

We ran the experiments for all of the hidden layer settings (16 runs) mentioned above. Training and validation set accuracies for Right Hand Generalized and Left Hand User Specific cases are shown in Figure 10 and Figure 11.

The other two cases show similar results. The figures show that validation set accuracies start to settle at around 15 units in the hidden layer. As a result, we chose to use this value for every subsequent experiment.

Table 2 shows training and validation set accuracies for the Generalized Gesture Recognition case. Confusion matrix for the Right Hand Generalized case with SVM for the 60-20-20 partition (Figure 8) is given in Table 3 to show which gestures are basically getting misclassified. Misclassified gesture pairs are marked in bold.

Looking at the confusion matrix, we can see that the classifier is mostly confusing Jab with Throw, Uppercut with Throw and Lift, Block with Lift. Confusion matrices for the left hand and for the right hand with ANN show similar results. This is normal at this stage of the experiment because these gestures have a lot of similarities in the way they were performed.

We used the test case “P7” (Figure 9) to examine our parameter selection and classifier accuracy for User Specific Gesture Recognition. Table 4 shows training and validation set accuracies for different datasets along with classifier training time.

From Table 5 we see that the gesture mix-ups are Throw with Jab, Block with Lift and Sway with Throw. Two of the gestures, Jab and Uppercut, were not recognized at all by the classifiers.

The gestures that are similar have not been classified properly in most of the cases. There can be two reasons behind this—one is that the current classifier parameters are not suitable for this type of datasets. The other one would be the dataset itself. Since the classifiers showed acceptable performance for the generalized cases (above 90% validation set accuracy), we are inclined to believe that the latter one is the reason behind such a poor performance. In particular, we believe that if we reduce the dimension of the dataset and keep only the data that contribute to most of the variations, we should be able to achieve better accuracy with current settings.

### 5.5. Dimensionality Reduction

As mentioned in Section 5.1, we calculated 115 features from the raw data. However, all of these features do not contribute well to every gesture in terms of variation in data. For example, most of the features calculated from sensors 15 and 16 would not be too useful to classify left-hand gestures because these two sensors were subject to very limited to no movement during the performance of those gestures. On the other hand, data from sensor 19 might be very useful to distinguish Sway because other gestures had very limited use of this sensor during the study. Therefore, we need to apply a proper dimensionality reduction technique to get the most out of our feature set. Principal Component Analysis (PCA) was our choice because its operating principles match with our requirements.

We applied PCA by using Weka’s [59] attribute selection tool which ranks the PCs in terms of their Eigenvalues and keeps only those that fall within the specified variation. Initially, we applied PCA on the entire feature dataset, ranked the principal component using Weka and kept those components which cumulatively attributed to 95% of the variation in the dataset. We followed this procedure for both the generalized and the user specific cases.

## 6. Result Analysis through Cross Validation and Testing

We applied 10-fold cross-validation on our validation sets. We will start with the generalized case and then move forward to the user specific case following the data partition hierarchy as shown in Figure 9 and Figure 10.

### 6.1. Generalized Gesture Recognition

Table 6lists training, cross-validation and test set accuracies of the classifiers over different partitions of the dataset for Generalized Gesture Recognition. It shows that the classifiers achieved near perfect accuracies on the test sets after PCA was applied. The confusion matrices for these datasets do not have interesting data to show because the accuracies are mostly perfect. Thus, we can conclude that the classifiers were able to classify almost all of the test set samples, with negligible misclassifications for the generalized case after PCA was applied.

### 6.2. User Specific Gesture Recognition

Similar to the previous case, we used 10-fold cross-validation to obtain validation set accuracies for User Specific Gesture Recognition. As seen in Table 7 below, the current configuration of the experiment yielded extremely poor validation accuracies for the user-specific test. Further investigation revealed that we overlooked a critical property of our study before deciding to use the same configuration for both the cases. This is discussed in detail below.

Every person performs gestures uniquely even after following the same set of instructions. During our study, some of the participants performed the gestures in quick successions whereas others were comparatively slower. As a result, while some of them might have performed five to six instances of the same gesture within the duration of one sample (4.5 to 5.5 s), others might have performed only 4–5 gestures within the same period. With this in mind, we made an assumption that keeping velocity based features (velocity and angular velocity) in the dataset confused the classifiers because every participant’s speed in performing the gestures varied significantly. This phenomenon basically made everyone’s gesture unique. To the classifiers, even two Jabs might look different because they were performed by different individuals which might have resulted in the severe performance drops.

To overcome this problem, we decided to remove the velocity-dependent features before applying the same experimental methodology. Table 8 below shows that in most of the cases, we achieved better test set accuracy than training or validation set accuracy. These training and cross-validation sets are actually similar to those under the generalized category as they have data from several participants.

The changes made by removing velocity and angular velocity-based features had a positive impact on classifier performance and we obtained near perfect to perfect accuracies for all of the cases. This proves that our assumption was true—velocity and angular velocity should not be included in datasets that are used to classify gestures performed by a specific individual. Based on this finding, we ran similar experiments on the generalized case after excluding the velocity-based features and found a slight decrease in accuracies.

## 7. Runtime Considerations

The recognition system was evaluated on an Intel Core i5 2.5 GHz processor with 8 GB RAM on a 64-bit Windows 8 operating system using the Weka machine learning tool. The recognition rates with SVM were 1.8 milliseconds per sample for the left-hand dataset, 0.65 milliseconds per sample for the right-hand dataset and 1.2 milliseconds per sample for the combined dataset. This gives us an average recognition rate of 1.22 milliseconds per sample. Although ANN had a higher training time, the recognition rate on the test sets was similar to that of SVM’s. Overall, this means we can have a thread that tests 500 samples per second; and at this rate, we should be able to achieve interactive rates with the recognition system. Thus, we can conclude that the classifiers were able to classify almost all of the test set samples quickly enough to allow interactive systems to be developed, with negligible misclassifications for the generalized case. It is important to note, that with these types of recognition rates, more complex compound gestures (gestures in parallel or in sequence) can be examined for interactivity.

## 8. Conclusions

From the results of our experiments, we can deduce that human gesture recognition is not a problem that can be solved using any out of the box classification system. Different scenarios demand different configurations of the experiment and different approach strategies for accurate classification.

We built a complete human gesture recognition system based on Support Vector Machine and Artificial Neural Network for six different gestures by collecting data from 11 participants. We explored two scenarios by organizing the same dataset in two different ways: Generalized Gesture Recognition, where we included data from every individual in our test sets to test our system’s performance on recognizing the gestures, regardless of who performed the gestures, and User Specific Gesture Recognition where we tested if our system can perform well when it is given a test dataset from a specific individual to mimic a real life use of the system.

Our experiments revealed that if we have a good set of features, it is easier to recognize random human gestures in general as compared to recognizing a set of gestures from an individual alone. While achieving very good accuracy for the former requires applying basic data preprocessing techniques and common dimensionality reduction methods that are most commonly used in the literature, achieving the same results in the latter scenario is trickier. It requires a good understanding of the dataset and proper feature selection methods. We achieved near perfect gesture recognition in the generalized case by following standard experimental methodologies such as feature extraction, standardization, cross-validation and dimensionality reduction using PCA. However, the same methodology performed poorly on the user specific case. To overcome this problem, we decided to exclude all velocity-based features from our feature set and then follow the same gesture recognition procedure as mentioned above. Eventually, we were able to achieve near perfect overall recognition rates on all of our datasets for this case.

## 9. Limitations and Future Work

Currently, one of the limitations of this system is the provision of an uninterrupted power supply. The IMUs can be powered by two different sources of energy. They can either be plugged into a computer or other devices to supply power from USB ports or they can run on battery power. The first one restricts mobility and limits portability, whereas the second one is not a reliable source of uninterrupted energy. Using rechargeable battery cells with higher energy capacity (mAh rating) may improve this situation, but it would make the whole system expensive. The sensors have a few flaws in their design which restricts worry-free handling. The power/reset button on these devices protrudes outward which makes it prone to being pressed if the user is not aware of its presence while performing gestures. We are also trying to design better cases or containers for the sensors so that they can be easily strapped to a participant’s body. Lastly, the relatively larger size of the sensors would most likely prevent researchers from using it for gesture recognition using fingers.

We can envision several possible future applications of our work. The velocity-based features can be used to perform a user validation study that would recognize different users based on the way they perform gestures. A very important application of our research would be industrial process automation where industrial robots can be trained to mimic human gestures to perform heavy lifting and do other tasks that are otherwise difficult and dangerous for humans. While similar systems have already been developed, they can mostly only perform specific, pre-designed tasks [42,43]. Other workers in those factories still have to perform the dangerous but more sophisticated tasks by hand. As a future task, our aim is to carry this research forward, develop a full-body gesture recognition system by incorporating more sensors and integrate it into a robotic system.

## Figures and Tables

**Table 1:** Number of samples in the gesture dataset.

Gesture	LH * Samples	RH * Samples	Combined (LH + RH)
Jab	102	92	194
Uppercut	105	96	201
Throw	105	96	201
Lift	95	86	181
Block	95	86	181
Sway	66	56	122
Total # of samples:	568	512	1080

* LH = Left Hand, RH = Right Hand.

**Table 2:** Training and validation set accuracies for different datasets (generalized category).

Dataset	Classifier					
	SVM			ANN		
	Training Time (s)	Training Accuracy (%)	Validation Accuracy (%)	Training Time (s)	Training Accuracy (%)	Validation Accuracy (%)
Left Hand	1.76	99.71	93.04	12.6	97.65	93.91
Right Hand	1.03	100	94.39	12.7	98.36	90.65

**Table 3:** Confusion matrix (in %) for validation set accuracy of Right Hand (60-20-20), SVM.

	Jab	Uppercut	Throw	Lift	Block	Sway
Jab	78.9	0	**21.1**	0	0	0
Uppercut	0	85.0	**10.0**	**5.0**	0	0
Throw	0	0	100	0	0	0
Lift	0	0	0	100	0	0
Block	0	0	0	**16.7**	83.3	0
Sway	0	0	0	0	0	100

**Table 4:** Training and validation set accuracies for different datasets (user specific category).

Dataset	Classifier					
	SVM			ANN		
	Training Time (s)	Training Accuracy (%)	Validation Accuracy (%)	Training Time (s)	Training Accuracy (%)	Validation Accuracy (%)
Left Hand	1.06	100	38.39	13.65	99.45	36.61
Right Hand	1.03	100	58.00	12.62	99.70	49.00

**Table 5:** Confusion matrix (in %) for validation set accuracy of Right Hand, ANN.

	Jab	Uppercut	Throw	Lift	Block	Sway
Jab	0.0	0	41.2	35.3	23.5	0
Uppercut	0	0.0	0	0	100	0
Throw	35.0	0	65.0	0	0	0
Lift	0	0	0	50.0	50.0	0
Block	10.0	5.2	0	35.0	50.0	5.0
Sway	0	0	33.3	0	0	66.7

**Table 6:** Training, validation and test set accuracies (Generalized, after PCA).

Partition	Classifier	Partition	Left Hand Accuracy (%)	Right Hand Accuracy (%)	Combined Accuracy (%)
60-20-20	SVM	Training	100	100	---
		CV	99.7	100	---
		**Test**	**99.1**	**99.0**	---
	ANN	Training	100	100	---
		CV	99.7	99.7	---
		**Test**	**99.1**	**99.0**	---
70-30	SVM	Training	100	100	99.9
		**Test**	**100**	**100**	**99.7**
	ANN	Training	100	100	99.9
		**Test**	**100**	**99.3**	**99.7**
60-40	SVM	Training	100	100	100
		**Test**	**99.5**	**100**	**100**
	ANN	Training	100	100	100
		**Test**	**99.0**	**100**	**100**

**Table 7:** Training and validation set accuracies (User Specific, after PCA).

Test Case	P1			
Classifier	SVM		ANN	
Accuracy →	Training	Validation	Training	Validation
Dataset ↓				
Left Hand	99.76	9.29	99.76	12.65
Right Hand	100	12.73	100	7.27
Combined	99.87	10	99.87	15.45

**Table 8:** Training, validation and test set accuracies (User Specific, PCA and velocity removal).

Test Case	Classifier	Partition	Left Hand Accuracy (%)	Right Hand Accuracy (%)	Combined Dataset Accuracy (%)
P1	SVM	Training	99.67	100	99.82
		Validation	98.26	100	100
		**Test**	**100**	**100**	**100**
	ANN	Training	99.67	99.63	99.82
		Validation	99.13	100	100
		**Test**	**100**	**100**	**100**
P7	SVM	Training	99.73	100	99.82
		Validation	99.75	90	86.79
		**Test**	**100**	**100**	**100**
	ANN	Training	99.45	100	99.86
		Validation	100	100	91.04
		**Test**	**100**	**100**	**100**
P11	SVM	Training	99.74	100	99.86
		Validation	89.01	88.61	88.82
		**Test**	**100**	**88.89**	**100**
	ANN	Training	99.74	100	99.86
		Validation	89.01	96.2	90
		**Test**	**98.33**	**100**	**99.12**

## Supplementary Materials

The dataset is available online at http://www.mdpi.com/1424-8220/16/5/605/s1.

## References

[1] Mitra S., Acharya T. (2007). Gesture Recognition: A Survey. IEEE Trans. Syst. Man Cybern. Part C Appl. Rev..

[2] Hofmann F.G., Heyer P., Hommel G. (1998). Velocity profile based recognition of dynamic gestures with discrete Hidden Markov Models. Gesture Sign Lang. Human-Comput. Interact..

[3] Moeslund T.B., Granum E. (2001). A Survey of Computer Vision-Based Human Motion Capture. Comput. Vis. Image Underst..

[4] Kim J.-H., Thang N.D., Kim T.-S. 3-D hand motion tracking and gesture recognition using a data glove. Proceedings of the IEEE International Symposium on Industrial Electronics (ISIE 2009).

[5] Zhang X., Chen X., Li Y., Lantz V., Wang K., Yang J. (2011). A framework for hand gesture recognition based on accelerometer and EMG sensors. IEEE Trans. Syst. Man Cybern. Part A Syst. Hum..

[6] Wang S., Yang J., Chen N., Chen X., Zhang Q. Human activity recognition with user-free accelerometers in the sensor networks. Proceedings of the 2005 International Conference on Neural Networks and Brain.

[7] Arsenault D. (2014). A Quaternion-Based Motion Tracking and Gesture Recognition System Using Wireless Inertial Sensors. Master Thesis.

[8] Song Y., Gu Y., Wang P., Liu Y., Li A. A Kinect based gesture recognition algorithm using GMM and HMM. Proceedings of the 2013 6th International Conference on Biomedical Engineering and Informatics.

[9] Schlömer T., Poppinga B., Henze N., Boll S. Gesture recognition with a Wii controller. Proceedings of the 2nd International Conference on Tangible and Embedded Interaction.

[10] Lementec J.-C., Bajcsy P. Recognition of arm gestures using multiple orientation sensors: gesture classification. Proceedings of the 7th International IEEE Conference on Intelligent Transportation Systems.

[11] Wu Y., Huang T.S. Vision-Based Gesture Recognition: A Review. Gesture-Based Communication in Human-Computer Interaction, Proceedings of the International Gesture Workshop (GW’99).

[12] Liu J., Zhong L., Wickramasuriya J., Vasudevan V. (2009). UWave: Accelerometer-based personalized gesture recognition and its applications. Pervasive Mob. Comput..

[13] Reifinger S., Wallhoff F., Ablassmeier M., Poitschke T., Rigoll G. (2007). Static and dynamic hand-gesture recognition for augmented reality applications. Human-Comput. Interact. Pt 3 Proc..

[14] Zhu C., Sheng W. (2011). Wearable sensor-based hand gesture and daily activity recognition for robot-assisted living. IEEE Trans. Syst. Man Cybern. Part A Syst. Hum..

[15] Gowing M., Ahmadi A., Destelle F., Monaghan D.S., O’Connor N.E., Moran K. (2014). Kinect *vs.* low-cost inertial sensing for gesture recognition. Lect. Notes Comput. Sci..

[16] Lyons K., Brashear H., Westeyn T., Kim J.S., Starner T. (2007). GART: The gesture and activity recognition toolkit. Human-Comput. Interact. Pt 3 Proc..

[17] Cooney M.D., Becker-Asano C., Kanda T., Alissandrakis A., Ishiguro H. Full-body gesture recognition using inertial sensors for playful interaction with small humanoid robot. Proceedings of the 2010 IEEE/RSJ International Conference on Intelligent Robots and Systems (IROS).

[18] Majoe D., Widmer L., Tschiemer P., Gutknecht J. Tai Chi Motion Recognition Using Wearable Sensors and Hidden Markov Model Method. http://info.ee.surrey.ac.uk/CCSR/EuroSSC/2009/poster-session/Majoe09_EuroSSC.pdf.

[19] Lementec J.-C., Bajcsy P., Kooper R., Lementec J.C. Recognition of arm gestures using multiple orientation sensors: repeatability assessment. Proceedings of the 7th International IEEE Conference on Intelligent Transportation Systems.

[20] Benbasat A.Y., Paradiso J.A. (2001). Compact, configurable inertial gesture recognition. CHI ’01 Ext. Abstr. Hum. Factors Comput. Syst..

[21] Brahem M.B., Ménélas B.A.J., Otis M.J.D. (2013). Use of a 3DOF accelerometer for foot tracking and gesture recognition in mobile HCI. Procedia Comput. Sci..

[22] Otten P., Kim J., Son S.H. (2015). A framework to automate assessment of upper-limb motor function impairment: A feasibility study. Sensors.

[23] Li P., Meziane R., Otis M.J.D., Ezzaidi H., Cardou P. A smart safety helmet using IMU and EEG sensors for worker fatigue detection. Proceedings of the 2014 IEEE International Symposium on Robotic and Sensors Environments (ROSE).

[24] Leelasawassuk T. Estimating Visual Attention from a Head Mounted IMU. Proceedings of the 2015 ACM International Symposium on Wearable Computers.

[25] Maes P., Darrell T., Blumberg B., Pentland A. (1997). The ALIVE system: Wireless, full-body interaction with autonomous agents. Multimed. Syst..

[26] Peng B., Qian G., Rajko S. View-invariant full-body gesture recognition via multilinear analysis of voxel data. Proceedings of the Third ACM/IEEE International Conference on Distributed Smart Cameras (ICDSC 2009).

[27] Peng B., Qian G., Rajko S. View-invariant full-body gesture recognition from video. Proceedings of the 19th International Conference on Pattern Recognition (ICPR 2008).

[28] Choi H.-R., Cho H.Y., Kim T.Y. Dynamically Weighted DTW for Dynamic Full-Body Gesture Recognition. https://www2.ia-engineers.org/iciae/index.php/icisip/icisip2015/paper/-viewFile/719/502.

[29] Kistler F., Sollfrank D., Bee N., André E. (2011). Full body gestures enhancing a game book for interactive story telling. Lect. Notes Comput. Sci..

[30] De Silva S., Barlow M. An Evaluation of DTW Approaches for Whole-of-Body Gesture Recognition. Proceedings of the 28th International BCS Human Computer Interaction Conference (HCI 2014).

[31] Roggen D., Calatroni A., Rossi M., Holleczek T., Kilian F., Tröster G., Lukowicz P., Bannach D., Pirkl G., Ferscha A. Collecting complex activity datasets in highly rich networked sensor environments. Proceedings of the 2010 Seventh International Conference on Networked Sensing Systems (INSS).

[32] Sagha H., Digumarti S.T., Chavarriaga R., Calatroni A., Roggen D., Tröster G. Benchmarking classification techniques using the Opportunity human activity dataset. Proceedings of the 2011 IEEE International Conference on Systems, Man, and Cybernetics (SMC).

[33] Kurz M., Hölzl G., Ferscha A., Calatroni A., Roggen D., Tröster G. Real-Time Transfer and Evaluation of Activity Recognition Capabilities in an Opportunistic System. http://citeseerx.ist.psu.edu/viewdoc/download?doi=10.1.1.417.461&rep=rep1&type=pdf.

[34] Ruffieux S., Lalanne D., Mugellini E., Khaled O.A., Ruffieux S., Mugellini E., Aboukhaled O., Kurosu M. (2014). A Survey of Datasets for Human Gesture Recognition.

[35] LaViola J.J. (2013). 3D Gestural Interaction: The State of the Field. ISRN Artif. Intell..

[36] Dam E.B., Koch M., Lillholm M. Quaternions, Interpolation and Animation. http://web.mit.edu/2.998/www/QuaternionReport1.pdf.

[37] Ullah S., Higgins H., Braem B., Latre B., Blondia C., Moerman I., Saleem S., Rahman Z., Kwak K.S. (2012). A comprehensive survey of wireless body area networks. J. Med. Syst..

[38] Whitehead A., Crampton N., Fox K., Johnston H. Sensor networks as video game input devices. Proceedings of the 2007 conference on Future Play.

[39] Welch G., Foxlin E. (2002). Motion tracking: No silver bullet, but a respectable arsenal. IEEE Comput. Graph. Appl..

[40] Hol J. Sensor Fusion and Calibration of Inertial Sensors, Vision, Ultra-Wideband and GPS. http://citeseerx.ist.psu.edu/viewdoc/summary?doi=10.1.1.394.9651.

[41] Kim A., Golnaraghi M. Initial calibration of an inertial measurement unit using an optical position tracking system. Proceedings of the Position Location and Navigation Symposium (PLANS 2004).

[42] Mukundan R. Quaternions : From Classical Mechanics to Computer Graphics, and Beyond. Proceedings of the 7th Asian Technology Conference in Mathematics.

[43] Vicci L. (2001). Quaternions and Rotations In 3-Space: The Algebra and Its Geometric Interpretation.

[44] Duda R.O., Hart P.E., Stork D.G. (2000). Pattern Classification.

[45] Ng A. (2012). Support Vector Machines. Machine Learning.

[46] Boser B.E., Guyon I.M., Vapnik V.N. A Training Algorithm for Optimal Margin Classifiers. Proceedings of the COLT 92 Proceedings of the Fifth Annual Workshop on Computational Learning Theory.

[47] Benvenuto N., Piazza F. (1992). On the complex backpropagation algorithm. IEEE Trans. Signal Process..

[48] Anantwar S.G., Shelke R.R. (2012). Simplified Approach of ANN : Strengths and Weakness. Int. J. Eng. Innov. Technol..

[49] Guyon I.M. (2006). Feature Extraction: Foundations and Applications.

[50] Wang W., Miguel A. (2014). The Role of Dimensionality Reduction in Classification. AAAI'14 Proceedings of the Twenty-Eighth AAAI Conference on Artificial Intelligence.

[51] Wold S., Esbensen K., Geladi P. (1987). Principal component analysis. Chemom. Intell. Lab. Syst..

[52] Arsenault D., Whitehead A. Wearable Sensor Networks for Motion Capture. Proceedings of the 2015 7th International Conference on Intelligent Technologies for Interactive Entertainment (INTETAIN).

[53] Unity, Unity—Game Engine. http://unity3d.com.

[54] InvenSense, MPU-6000/6050 Six-Axis MEMS Motion Tracking Devices. http://www.invensense.com/products/motion-tracking/6-axis.

[55] Hibbeler R.C. (2009). Engineering Mechanics.

[56] Diebel J. (2006). Representing attitude: Euler angles, unit quaternions, and rotation vectors. Matrix.

[57] Hsu C.-W., Chang C.-C. (2008). A Practical Guide to Support Vector Classification. BJU Int..

[58] Bishop C.M. (1995). Neural Networks for Pattern Recognition.

[59] Hall M., Frank E., Holmes G., Pfahringer B., Reutemann P., Witten I.H. (2009). The WEKA Data Mining Software: An Update. ACM SIGKDD Explor. Newslett..

